# Comparison of metal-carbenoid reactions of β-2-five-membered heteroaryl substituted α,β-unsaturated ketones

**DOI:** 10.55730/1300-0527.3625

**Published:** 2023-10-11

**Authors:** Kumsal EROĞLU, Olcay ANAÇ, Füsun Şeyma KIŞKAN

**Affiliations:** Department of Chemistry, Faculty of Science and Letters, İstanbul Technical University, İstanbul, Turkiye

**Keywords:** Metal-carbenoid, diazocarbonyl, dihydrofuran, insertion, heteroaryl

## Abstract

In this study, *β*-2-heteroaryl substituted (*N*-methyl 2-pyrrolyl, 2-thiophenyl, 2-furyl) *α*,*β*-unsaturated ketones were reacted with two *α*-diazo carbonyl compounds that had different characteristics (dimethyl diazo malonate and 1-diazo-1-phenyl-propane-2-one) in the presence of both copper and rhodium catalysts. In the case of reactions with *N*-methyl 2-pyrrolyl *α*,*β*-unsaturated ketones, the major product was the insertion derivative. However, in the reactions of 2-thiophenyl and 2-furyl *α*,*β*-unsaturated ketones with dimethyl diazomalonate (acceptor-acceptor disubstituted), only dihydrofuran products were formed over carbonyl ylides. When 2-thiophenyl and 2-furyl *α*,*β*-unsaturated ketones were reacted with 1-diazo-1-phenyl-propane-2-one (donor-acceptor disubstituted), 1-phenylpropane-1,2-dione was obtained under our reaction conditions.

## 1. Introduction

*α*-Diazocarbonyl compounds are a versatile class of compounds in organic synthesis. They are useful intermediates due to their decompositions under various conditions, especially in the presence of transition metal catalysts, to obtain metal-carbenoids [1–3].

**Scheme 1:**
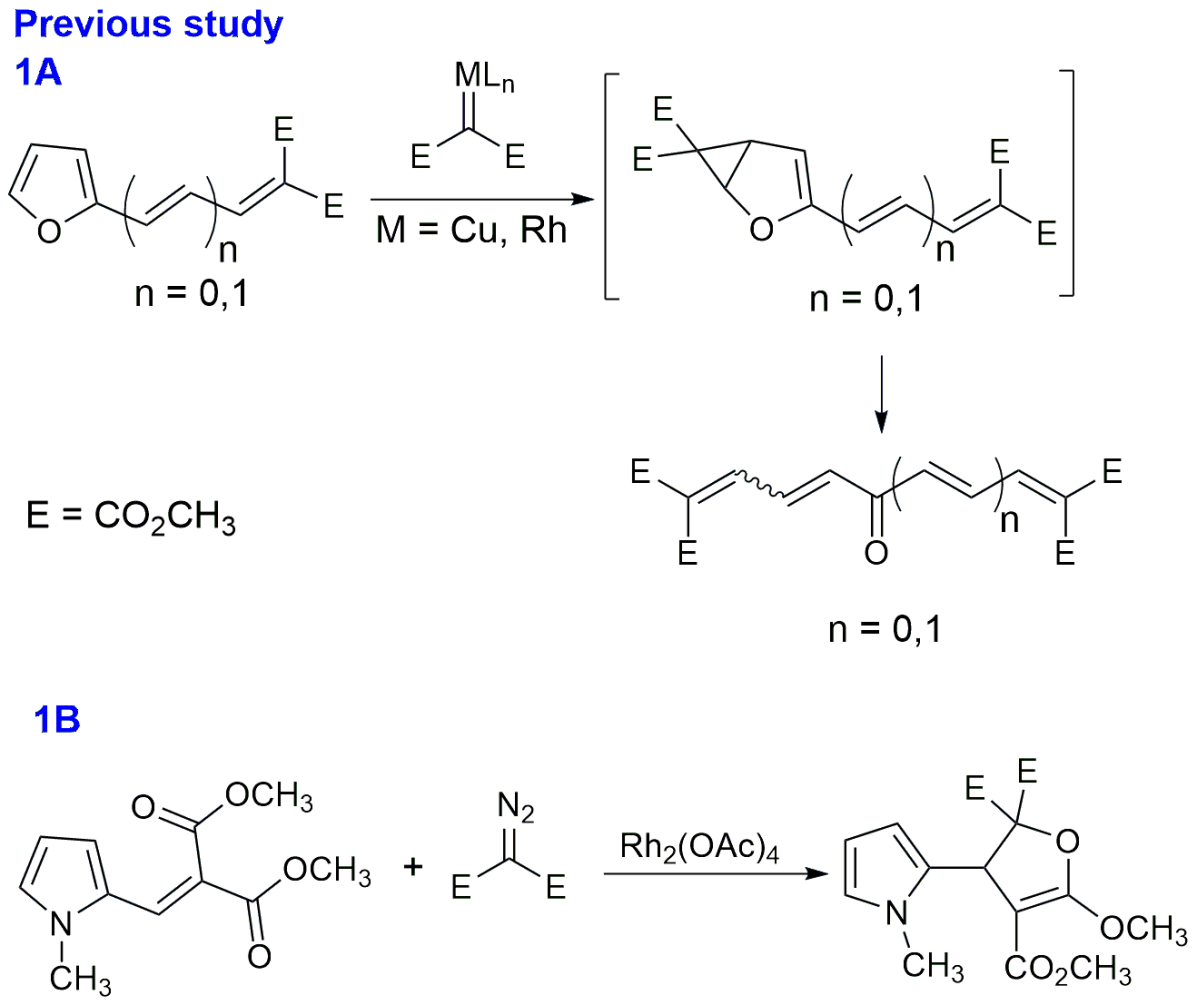
Reactions of furyl methylene/allylidene malonates and *N*-methyl 2-pyrrolylmethylene dimalonate with dimethyl diazomalonate [24].

Biological and medicinal chemistry studies with heterocyclic compounds have increased in importance to produce many naturally occurring bioactive compounds and synthetic pharmaceuticals [4,5]. Recently, many synthetic methods involving aromatic heterocycles (especially five-membered) and metal-carbenoids have been developed [6]. Heterocycles containing oxygen, sulfur, and nitrogen atoms are electron-rich compounds that can easily undergo reactions with electrophilic metal-carbenoids [7–10]. Although metal-carbenoid reactions for the modification of pyrrole cores have been reported previously [11–16], the literature offers a limited number of examples of reactions with thiophene/furan derivatives [17–23]. According to the current literature on the metal-carbenoid reactions of 2-substituted heteroaryl compounds, variation of the substituents may alter the reaction pathway deriving from either the heterocyclic core or the substituent [7,20]. In our continuous efforts for the metal-carbenoid transformations of 2-substituted five-membered heterocycles [24], we found that furans with only 2-ene/diene-diester functions smoothly gave the corresponding polymethoxy carboxylate-substituted oxo-polyenes over furyl unraveling in good yields (Schemes 1A and 1B). Continuing our research, this study reports the results of the reactions of *β*-2-heteroaryl *α*,*β*-unsaturated ketone with *α*-diazo compounds. We compared the reaction mechanisms of *N*-methyl 2-pyrrolyl, 2-thiophenyl, and 2-furyl derivatives (**1**–**3**) that contain the same ene-methylketo function at their 2-positions with two different types of diazo carbonyl compounds (**4** and **5**) under two catalytic conditions (x and y).

## 2. Experimental

### 2.1. General information

Reactions of diazo compounds and heteroaryl carbonyls were carried out under nitrogen atmosphere. A rotary evaporator equipped with a water condenser and attached to a vacuum system was used to concentrate in vacuo. All solvents and reactants were commercially available. Dimethyl diazo malonate (**4**) [25] and 1-diazo-1-phenyl-propane-2-one (**5**) [26] were prepared by literature procedures. ^1^H NMR and ^13^C NMR spectra in CDCl_3_ were recorded on an Agilent VNMRS (Agilent Technologies, Santa Clara, CA, USA) at 500 and 125 MHz, respectively. Chemical shifts (*δ*) are reported in ppm downfield from tetramethyl silane at ambient temperature. GC-MS analyses were performed on a Thermo Finnigan trace DSQ instrument (Thermo Fisher, Waltham, MA, USA) equipped with a flame ionization detector. A 5% phenyl polyphenylene-siloxane capillary column (TR-5MS, Thermo Fisher) was used with helium as the carrier gas. The temperature program was as follows: start at 100 °C, then 5 min isothermal, ramp at 20 °C/min; final 290 °C, and then 10 min isothermal. Retention times (*t*_R_) are given in minutes. HR-MS: Agilent 6230-B TOF LC/MS in m/z.

### 2.2. Reactions of substrates and diazo compounds

For condition x, to a solution of CuCl (0.15 mmol), AgSbF_6_ (0.15 mmol), (−)-2,2′-isopropylidene bis[(4*S*)-4-phenyl-2-oxazoline] (0.15 mmol), and a molecular sieve (4 Å) were added and the mixture was refluxed for 1 h. A heteroaryl carbonyl compound (**1**–**3**) (1.7 mmol) was added to the reaction mixture and the mixture was stirred for 5 min. A solution of diazo compound (1.7 mmol) in benzene (3 mL) was added to this mixture over 2.5 h under N_2_ atmosphere. When the IR spectrum of the reaction mixture indicated the total consumption of the diazo compound (absence of the characteristic diazo band), the mixture was filtered, evaporated, and purified by column chromatography or preparative thin-layer chromatography.

For condition y, to a solution of the heteroaryl carbonyl compound (**1**–**3**) (2 mmol) in solvent (benzene if the diazo compound was dimethyl diazomalonate or CH_2_Cl_2_ if the diazo compound was 1-diazo-1-phenylpropane-2-one) (20 mL) was added Rh_2_(OAc)_4_ (0.01 mmol), and the mixture was heated at reflux. A solution of diazo compound (1.4 mmol) in solvent (2 mL) was added to this solution over 2.5 h under N_2_ atmosphere. When the IR spectrum of the reaction mixture indicated the total consumption of the diazo compound (absence of the characteristic diazo band), the mixture was filtered, evaporated, and purified by column chromatography or preparative thin-layer chromatography.

#### 2.2.1. Dimethyl 5-methyl-3-(1-methyl-1H-pyrrole-2-yl)furan-2,2(3H)-dicarboxylate (6_1–4_)

**6_1_**_–_**_4_** was purified by silica column chromatography with hexane and ethyl acetate (1:1) as an eluent. Orange-brownish oil; yield 25% (with condition x); IR ν_max_ (CH_2_Cl_2_): 2957, 2924, 1742, 1435, 1284, 1217, 1187, 915, 719 cm^−1^; ^1^H NMR (500 MHz, CDCl_3_) *δ* 6.52 (t, *J* = 2.50 Hz, 1H, pyrrolyl-*H*), 6.00 (t, *J* = 3.05 Hz, 1H, pyrrolyl-*H*), 5.94 (dd, *J* = 3.65/1.70 Hz, 1H, pyrrolyl-*H*), 5.15 (t, *J* = 2.05 Hz, 1H, C*H*-CH=C), 4.67 (dd, *J* = 2.30/1.25 Hz, 1H, C*H*=C-CH_3_), 3.86 (s, 3H, CO_2_C*H*_3_), 3.63 (s, 3H, CO_2_C*H*_3_), 3.34 (s, 3H, N-C*H*_3_), 1.98 (dd, *J* = 1.95/1.25 Hz, 3H, CH=C-C*H*_3_); ^13^C NMR (125 MHz, CDCl_3_) *δ* 168.6 (*C*O_2_CH_3_), 166.9 (*C*O_2_CH_3_), 154.0 (CH=*C*-CH_3_), 129.5 (*C*_pyrrolyl_), 122.7 (*C*_pyrrolyl_), 108.8 (*C*_pyrrolyl_), 106.9 (*C*_pyrrolyl_), 98.1 (*C*=C-CH_3_), 91.7 (*C*(CO_2_CH_3_)_2_), 53.5 (CO_2_*C*H_3_), 52.6 (CO_2_*C*H_3_), 46.6 (*C*H-CH=C), 33.8 (N-*C*H_3_), 13.3 (CH=C-*C*H_3_); *t*_R_: 12.95; EI-MS (m/z): 221 (M^+^-CH_3_, 57), 162 (100), 118 (66), 91 (19), 59 (10); HRMS: Calcd for C_14_H_18_NO_5_ [M+H]^+^ 280.1179, found 280.1185.

#### 2.2.2. Dimethyl 5-methyl-3-(thiophene-2-yl)furan-2,2(3H)-dicarboxylate (6_2–4_)

**6_2_**_–_**_4_** was purified by preparative silica thin-layer chromatography with hexane and ethyl acetate (7:1) as a mobile phase. Dark yellow oil, yield 92% (with condition x); IR ν_max_ (CH_2_Cl_2_): 2927, 1744, 1433, 1287 cm^−1^; ^1^H NMR (500 MHz, CDCl_3_) *δ* 7.20 (dd, *J* = 5.00/1.30 Hz, 1H, thiophenyl-*H*), 6.94 (dd, *J* = 5.00/3.50 Hz, 1H, thiophenyl-*H*), 6.93–6.91 (m, 1H, thiophenyl-*H*), 5.28–5.27 (m, 1H, C*H*-CH=C), 4.84–4.83 (m, 1H, C*H*=C-CH_3_), 3.87 (s, 3H, CO_2_C*H*_3_), 3.37 (s, 3H, CO_2_C*H*_3_), 2.01 (dd, *J* = 1.80/1.35 Hz, 3H, CH=C-C*H*_3_); ^13^C NMR (125 MHz, CDCl_3_) *δ* 168.3 (*C*O_2_CH_3_), 166.2 (*C*O_2_CH_3_), 155.4 (CH=*C*-CH_3_), 141.9 (*C*_thiophenyl_), 126.8 (*C*_thiophenyl_), 126.7 (*C*_thiophenyl_), 125.2 (*C*_thiophenyl_), 99.4 (*C*H=C-CH_3_), 92.2 (*C*(CO_2_CH_3_)_2_), 53.6 (CO_2_*C*H_3_), 52.4 (CO_2_*C*H_3_), 49.7 (*C*H-CH=C), 13.4 (CH=C-C*H*_3_); *t*_R_: 12.00; EI-MS (m/z): 282 (M^+^, 35), 222 (100), 191 (80), 164 (61), 151 (48), 135 (57), 91 (39), 59 (27); HRMS: Calcd for C_13_H_15_O_5_S [M+H]^+^ 283.0635, found 283.0640.

#### 2.2.3. Dimethyl 5′-methyl-[2,3′-bifuran]-2′,2′(3′H)-dicarboxylate (6_3–4_)

**6_3_**_–_**_4_** was purified by preparative silica thin-layer chromatography with hexane and ethyl acetate (1:2) as a mobile phase. Yellow oil; yield 88% (with condition x); IR ν_max_ (CH_2_Cl_2_): 2968, 2924, 2855, 1744, 1436, 1237 cm^−1^; ^1^H NMR (500 MHz, CDCl_3_) *δ* 7.34 (d, *J* = 1.00 Hz, 1H, furyl-*H*), 6.30 (dd, *J* = 3.05/1.90 Hz, 1H, furyl-*H*), 6.18 (d, *J* = 3.15 Hz, 1H, furyl-*H*), 5.14 (t, *J* = 1.90 Hz, 1H, C*H*-CH=C), 4.72–4.71 (m, 1H, CH-C*H*=C), 3.87 (s, 3H, CO_2_C*H*_3_), 3.47 (s, 3H, CO_2_C*H*_3_), 1.99 (bs, 3H, CH=C-C*H*_3_); ^13^C NMR (125 MHz, CDCl_3_) *δ* 168.0 (*C*O_2_CH_3_), 166.5 (*C*O_2_CH_3_), 155.3 (*C*_furyl_), 152.1 (CH=*C*-CH_3_), 142.3 (*C*_furyl_), 110.5 (*C*_furyl_), 108.4 (*C*_furyl_), 96.6 (CH-*C*H=C), 90.9 (*C*(CO_2_CH_3_)_2_), 53.6 (CO_2_*C*H_3_), 52.8 (CO_2_*C*H_3_), 48.7 (*C*H-CH=C), 13.4 (CH=C-*C*H_3_); *t*_R_: 10.85; EI-MS (m/z): 266 (M^+^, 1), 238 (15), 207 (72), 174 (100), 148 (45), 119 (32), 105 (24), 91 (85), 65 (27), 59 (25); HRMS: Calcd for C_13_H_15_O_6_ [M+H]^+^ 267.0863, found 267.0875.

#### 2.2.4. Dimethyl (E)-2-(1-methyl-5-(3-oxobut-1-ene-1-yl)-1H-pyrrole-2-yl)malonate (7_1–4_)

**7_1_**_–_**_4_** and **8_1_**_–_**_4_** were purified by silica column chromatography with hexane and ethyl acetate (1:1) from the crude mixture. From the mixture of **7_1_**_–_**_4_** and **8_1_**_–_**_4_** (2:1, respectively): ^1^H NMR (500 MHz, CDCl_3_) *δ* 7.42 (d, *J* = 15.75 Hz, 1H, C*H*=CH-CO), 6.91 (d, *J* = 1.7 Hz, 1H, pyrrolyl-*H*), 6.73 (d, *J* = 1.7 Hz, 1H, pyrrolyl-*H*), 6.52 (d, *J* = 15.75 Hz, 1H, CH=C*H*-CO), 4.57 (s, 1H, C*H*(CO_2_CH_3_)_2_), 3.77 (s, 6H, CO_2_C*H*_3_), 3.70 (s, 3H, N-C*H*_3_), 2.30 (s, 3H, COC*H*_3_); ^13^C NMR (125 MHz, CDCl_3_) *δ* 197.6 (*C*=O), 167.2 (*C*O_2_CH_3_), 130.5 (*C*H=CH-CO), 130.1 (*C*_pyrrolyl_), 129.4 (*C*_pyrrolyl_), 127.0 (*C*_pyrrolyl_), 115.6 (*C*_pyrrolyl_), 112.5 (CH=*C*HCO), 50.4 (*C*H(CO_2_CH_3_)_2_), 31.3 (N-*C*H_3_), 28.3 (CO*C*H_3_); *t*_R:_ 12.26; EI-MS (m/z): 279 (M^+^, 13), 220 (80), 188 (39), 160 (42), 132 (100), 108 (49), 59 (18).

#### 2.2.5. (E)-4-(1-Methyl-5-(2-oxo-1-phenyl propyl)-1H-pyrrole-2-yl)but-3-ene-2-one (7_1–5_)

**7_1_**_–_**_5_** and **8_1_**_–_**_5_** were purified by silica column chromatography with hexane and ethyl acetate (1:2) from the crude mixture. From the mixture of **7_1_**_–_**_5_** and **8_1_**_–_**_5_** (1.4:1, respectively): IR ν_max_ (CH_2_Cl_2_) (cm^−1^) 2924, 2857, 1709, 1451, 1358 cm^−1^; ^1^H NMR (500 MHz, CDCl_3_) *δ* 7.47 (d, *J* = 15.65 Hz, 1H, C*H*=CH-CO), 7.38–7.28 (m, 3H, Ar-*H*), 7.19–7.16 (m, 2H, Ar-*H*), 6.70 (d, *J* = 4.10 Hz, 1H, pyrrolyl-*H*), 6.53 (d, *J* = 15.65 Hz, 1H, CH=C*H*-CO), 5.99 (d, *J* = 4.10 Hz, 1H, pyrrolyl-*H*), 5.07 (s, 1H, C*H*(CO_2_CH_3_)), 3.47 (s, 3H, N-C*H*_3_), 2.30 (s, 3H, COC*H*_3_), 2.28 (s, 3H, COC*H*_3_); ^13^C NMR (125 MHz, CDCl_3_) *δ* 204.3 (*C*=O), 197.6 (*C*=O), 135.9 (*C*_Ar_), 130.3 (*C*_Ar_), 128.9 (*C*_Ar_), 126.8 (*C*_Ar_), 122.2 (*C*_pyrrolyl_), 121.8 (*C*H=CHCO), 121.6 (*C*_pyrrolyl_), 112.6 (CH=*C*HCO), 111.5 (*C*_pyrrolyl_), 109.7 (*C*_pyrrolyl_), 58.0 (*C*H(CO_2_CH_3_)), 34.4 (N-*C*H_3_), 29.6 (CO*C*H_3_), 28.7 (CO*C*H_3_); EI-MS (m/z): 281 (M^+^, 8), 238 (M^+^-COCH_3_, 100), 194 (37), 91 (8), 77 (10).

#### 2.2.6. (E)-4-(1-Methyl-4-(2-oxo-1-phenylpropyl)-1H-pyrrole-2-yl)but-3-ene-2-one (8_1–5_)

From the mixture of **7_1_**_–_**_5_** and **8_1_**_–_**_5_** (1.4:1, respectively): ^1^H NMR (500 MHz, CDCl_3_) *δ* 7.40 (d, *J* = 15.70 Hz, 1H, C*H*=CH-CO), 7.38–7.28 (m, 3H, Ar -*H*), 7.19–7.16 (m, 2H, Ar-*H*), 6.67 (d, *J* = 1.80 Hz, 1H, pyrrolyl-*H*), 6.56 (d, *J* = 1.80 Hz, 1H, pyrrolyl-*H*), 6.46 (d, *J* = 15.70 Hz, 1H, CH=C*H*-CO), 4.92 (s, 1H, C*H*(CO_2_CH_3_)), 3.67 (s, 3H, N-C*H*_3_), 2.29 (s, 3H, COC*H*_3_), 2.21 (s, 3H, COC*H*_3_); ^13^C NMR (125 MHz, CDCl_3_) *δ* 206.5 (*C*=O), 197.6 (*C*=O), 138.7 (*C*_Ar_), 130.6 (*C*_Ar_), 129.0 (*C*_Ar_), 128.6 (*C*_pyrrolyl_), 127.9 (*C*_pyrrolyl_), 127.3 (*C*_Ar_), 121.7 (*C*H=CHCO), 112.4 (CH=*C*HCO), 110.9 (*C*_pyrrolyl_), 63.7 (*C*H(CO_2_CH_3_)), 31.1 (N-*C*H_3_), 29.3 (CO*C*H_3_), 28.3 (CO*C*H_3_); EI-MS (m/z): 281 (M^+^, 9), 238 (M^+^-COCH_3_, 100), 194 (46), 91 (8), 77 (18).

## 3. Results and discussion

In this study, we synthesized *β*-2-heteroaryl *α*,*β*-unsaturated ketone compounds (**1**–**3**) according to the literature [27]. Two diazo carbonyl compounds, dimethyl diazomalonate (**4**) (acceptor-acceptor (A-A) disubstituted) and 1-diazo-1-phenyl-propane-2-one (**5**) (donor-acceptor (D-A) disubstituted), were reacted with synthesized *β*-2-heteroaryl *α*,*β*-unsaturated ketone compounds (**1**–**3**) under two different catalytic conditions. The results are summarized in Tables 1 and 2.

First of all, (*E*)-4-(1-methyl-1*H*-pyrrole-2-yl)but-3-en-2-one (**1**) and *α*-diazocarbonyl compounds (**4**, **5**) were reacted under two different catalytic conditions (Table 1). In most of our previous studies, we used copper(II) acetylacetonate [Cu(acac)_2_] as a catalyst in metal-carbenoid reactions [24,28,29]. For this reason, we first tried Cu(acac)_2_ in the reaction of compound **1** with dimethyl diazomalonate (**4**). However, we did not encounter any product except carbene dimer. Therefore, we planned to repeat the reactions with different catalysts in pursuit of the formation of possible products.

Zhou et al. reported that [1,5]- or [1,7]-ring closure products can be obtained from ylide intermediate under CuCl/AgSbF_6_/ligand catalytic conditions in a diastereo-controlled way [30]. Accordingly, we tried to use CuCl/AgSbF_6_/ligand as a catalyst (condition x) in the reaction of **1** and **4**. In this attempt, we obtained an insertion product to the pyrrole ring as a major product and a dihydrofuran derivative as a minor product (Table 1, entry *i*).

As is known, Rh-complexes have become the most common catalysts in diazo reactions, especially C-H insertion reactions [31–35]. Therefore, we needed to repeat the same reaction of **1** and **4** with Rh_2_(OAc)_4_ (condition y) to search for the probable change in the product distribution. It was observed that the yield of dihydrofuran (**6_1_**_–_**_4_**) decreased. As expected, insertion products (**7_1_**_–_**_4_**, **8_1_**_–_**_4_**) to the pyrrole ring were formed mainly depending on the decreasing steric probabilities.

In our previous study (Scheme 1B) [24], only the dihydrofuran product was obtained from the reaction of *N*-methyl 2-pyrrolylmethylydene malonate and dimethyl diazomalonate (**2**) with the Rh_2_(OAc)_4_ catalyst. The mechanism of formation of dihydrofuran is initiated by the [1,5]-electrocyclic ring closure of the corresponding carbonyl ylide intermediate, derived from the electron-deficient metal-carbenoid and one of the ester carbonyl oxygens of the malonate function (Scheme 1B) or the *α*,*β*-unsaturated keto carbonyl oxygen function (Table 1, compound **6**). However, the main insertion mechanism (in Table 1, compounds **7** and **8**) preferred the attack of the frontier metal-carbenoid to the pyrrole ring instead of the carbonyl oxygen under the same conditions.

As another attempt, reactions with 1-diazo-1-phenyl-propane-2-one (**5**) (donor-acceptor disubstituted) were also carried out under the same two conditions (x and y) with **1** (Table 1, entries *iii* and *iv*). Interestingly, while no dihydrofuran derivative was formed, sterically more possible insertion products (**7_1_**_–_**_5_** and **8_1_**_–_**_5_**) into the pyrrole ring were observed.

We also needed to investigate the reactions of *β*-2-thiophenyl methyleneketone (**2**) and *β*-2-furyl methyleneketone (**3**). The results of reactions **2** and **3** with diazo compounds **4** and **5** under catalytic conditions are summarized in Table 2.

**Scheme 2:**
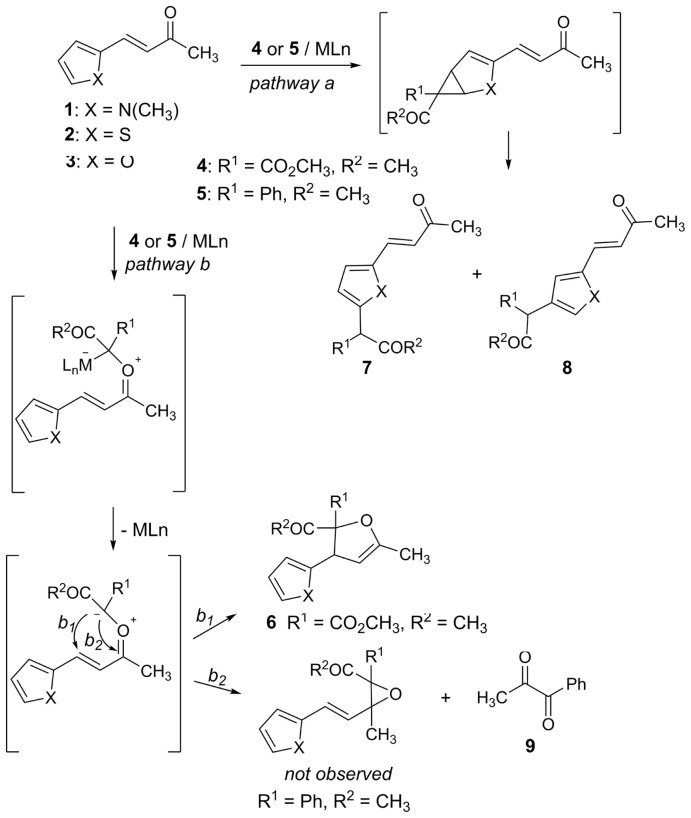
Heteroaryl C-H insertion of carbenoid (*path a*) [36] and electrocyclic [1,5]- or [1,3]-ring closure reactions of carbonyl ylide (*paths b_1_* and *b_2_*) [7,37].

No [1+1] products were observed between **2** or **3** and carbenoid **5** having a donor-acceptor character (Table 2, entries *iii*, *iv*, *vii*, and *viii*). Instead, a decomposition product (**9**) was obtained from these attempts (Scheme 2). However, corresponding dihydrofurans (**6_2_**_–_**_4_** and **6_3_**_–_**_4_**) were obtained as sole products from the reactions of dimethyl diazo malonate (**4**) with compounds **2** and **3**.

All products were obtained by two general mechanism pathways over the heteroaryl function (*path a*)/carbonyl oxygen (*paths b_1_* and *b_2_*) (Scheme 2).

As is known, the pyrrole ring is the most reactive species in electrophilic substitution according to thiophene and furan. Recent studies [38–40] have shown that the relative reactivities and regioselectivities of heteroaryl derivatives towards electrophiles are mostly variable, depending on the positions and nature of the substituents and also the types of electrophiles. When we performed the reactions of the *N*-methyl 2-pyrrolyl derivative (**1**) and diazo compounds (**4**, **5**), insertion products (**7**, **8**) to the pyrrole core were dominant (Table 1; Scheme 2, *path a*). The reactivity of the pyrrole core of **1** was effective for this chemoselective reaction. On the contrary, a 2-thiophenyl or 2-furyl-insertion product was not observed in the reactions with **2** and **3** (Table 2; Scheme 2, *path b*). The sole products from **2** and **3** with dimethyl diazomalonate (**4**) were dihydrofurans (**6**) formed via [1,5]-ring closure reactions over carbonyl ylide-intermediates (chemoselective reactions) (Scheme 2, *path b_1_*).

In our previous study, polyenone formations were observed by opening reactions of corresponding furan rings from *β*-2-furyl ene/diene-diesters and dimethyl diazomalonate under catalytic conditions (Scheme 1A). However, the carbonyl ylide was formed from the *β*-2-furyl ene-ketone function with dimethyl diazomalonate to yield compound **6** (Scheme 2, *path b_1_*). Therefore, different chemoselectivities were observed in the catalytic reactions of *β*-2-furyl ene/diene-diesters and *β*-2-furyl ene-ketone with dimethyl diazomalonate (**4**).

On the other hand, dimethyl diazomalonate (**4**) with acceptor-acceptor substituents directed the reaction to *path b_1_* (Scheme 2), but 1-diazo-1-phenyl-2-propanone (**5**) with donor-acceptor substituents led to *path b_2_* (Scheme 2).

**Scheme 3:**
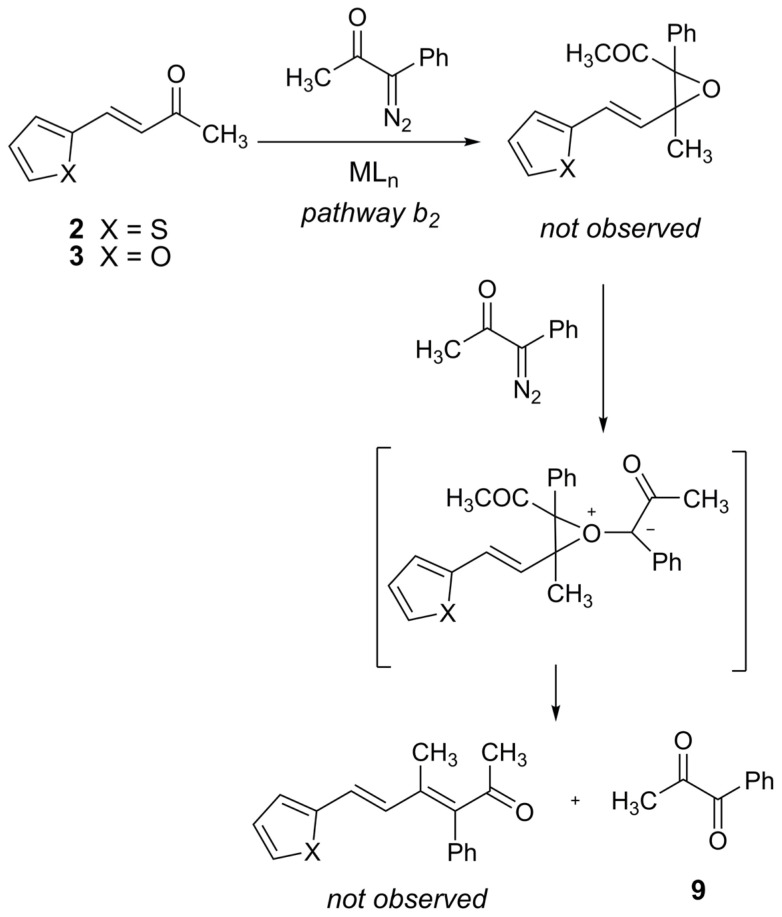
Formation of 1-phenylpropane-1,2-dione (**9**).

The only detectable compound was 1-phenylpropane-1,2-dione (**9**) in the reactions of **2** and **3** with 1-diazo-1-phenyl-2-propanone (**5**) (Scheme 2, *path b_2_*). According to the literature, 1,2-dione compounds such as 1-phenylpropane-1,2-dione (**9**) were formed by oxygen transfer to the diazo compound in a catalytic environment [41–43]. Yu et al. [44] also synthesized dimethyl 2-oxomalonate by Cu-catalyzed deoxygenation of epoxide with dimethyl diazomalonate. Therefore, we propose that under our reaction conditions, both the formation and decomposition of an epoxide might have occurred successively (Scheme 3).

## 4. Conclusion

In this study, we performed the reactions of *β*-2-heteroaryl substituted *α*,*β*-unsaturated ketones (**1**–**3**) with *α*-diazo compounds (**4**, **5**) under catalytic conditions (x, y). Insertion products (**7_1_**_–_**_4_** and **8_1_**_–_**_4_**) in the pyrrole ring were obtained as the main product from the reactions of *β*-*N*-methyl 2-pyrrolyl *α*,*β*-unsaturated ketone (**1**) with dimethyl diazomalonate (**4**) under both catalytic conditions. The dihydrofuran derivative (**6_1_**_–_**_4_**) was also found as a byproduct in these reactions. In the reactions of *β*-2-thiophenyl and *β*-2-furyl *α*,*β*-unsaturated ketones (**2** and **3**) with dimethyl diazomalonate (**4**), no insertion product was observed. Instead, dihydrofurans (**6_2_**_–_**_4_** and **6_3_**_–_**_4_**) were obtained as single products.

When dimethyl diazomalonate (**4**) was replaced with 1-diazo-1-phenyl-2-propanone (**5**) as a carbene source, no dihydrofuran was formed in any reaction, but 1-phenyl-propane-1,2-dione (**9**) could be obtained. In conclusion, the reactions of three *β*-2-heteroaryl substituted *α*,*β*-unsaturated ketones and two metal-carbenoids with acceptor-acceptor and donor-acceptor groups were carried out under the same conditions. While acceptor-acceptor groups containing the metal-carbenoid preferred to attack the *N*-methyl 2-pyrrolyl ring, the same carbenoid did not react with the 2-thiophenyl or 2-furyl rings.

## Figures and Tables

**Table 1:** Reactions of (*E*)-4-(1-methyl-1*H*-pyrrole-2-yl)but-3-ene-2-one (**1**) with α-diazocarbonyl compounds.

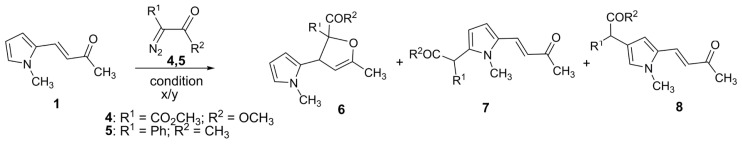					
Entry	Diazo comp.	Condition^a^	**6** ratio %^b^ (yield)^c^	**7** ratio %^b^ (yield)^c^	**8** ratio %^b^ (yield)^c^
*i*	**4**	x	**6_1–4_** 29.5 (25)	**7_1–4_** 70.5	-
*ii*	**4**	y	**6_1–4_** 18 (10.3)	**7_1–4_** 66.0	**8_1–4_** 15.5
*iii*	**5**	x	-	**7_1–5_** trace	-
*iv*	**5**	y	-	**7_1–5_** + **8_1–5_** 54.5 + 45.5	**7_1–5_** + **8_1–5_** 54.5 + 45.5

a Condition x: CuCl (0.15 mmol)/AgSbF_6_ (0.15 mmol)/isopropylidenebis(4*S*)-4-phenyl-2-oxazoline (0.15 mmol), compound **1**–**3** (1.7 mmol), and diazo compound (1.7 mmol); condition y: Compound **1**–**3** (2 mmol), Rh_2_(OAc)_4_ (0.01 mmol), and diazo compound (1.4 mmol).

b Relative product ratio based on ^1^H NMR analysis of crude reaction mixture.

c Isolated yield of chromatographically pure substances.

**Table 2:** Reactions of compounds **2** and **3** with α-diazocarbonyl compounds (**4** and **5**).

Entry	Heteroaryl	Diazo comp.	Condition^a^	Dihydrofuran 6 (yield)^b^
*i*	**2**	**4**	x	**6_2–4_** (92)
*ii*	**2**	**4**	y	**6_2–4_** (60)
*iii*	**2**	**5**	x	**-**
*iv*	**2**	**5**	y	**-**
*v*	**3**	**4**	x	**6_3–4_** (88)
*vi*	**3**	**4**	y	**6_3–4_** (40)
*vii*	**3**	**5**	x	**-**
*viii*	**3**	**5**	y	**-**

a Condition x: CuCl (0.15 mmol)/AgSbF_6_ (0.15 mmol)/isopropylidenebis(4*S*)-4-phenyl-2-oxazoline (0.15 mmol), compound **1**–**3** (1.7 mmol), and diazo compound (1.7 mmol); condition y: Compound **1**–**3** (2 mmol), Rh_2_(OAc)_4_ (0.01 mmol), and diazo compound (1.4 mmol).

b Isolated yield of chromatographically pure substances.
